# Factors Influencing Clinical Competence Among Nursing Students: Exploring Emotional Intelligence and Sleep Quality

**DOI:** 10.17533/udea.iee.v43n2e14

**Published:** 2025-07-25

**Authors:** Priyanshi Dixit, Anjali Rathee, Surya Kant Tiwari, Uma Phalswal, Smita Das

**Affiliations:** 1 . College of Nursing, Vardhman Mahavir Medical College, Safdarjung Hospital, New Delhi, India. Email: priyanshidixit17@gmail.com. https://orcid.org/0000-0001-5130-40371 Vardhman Mahavir Medical College College of Nursing Vardhman Mahavir Medical College Safdarjung Hospital New Delhi India priyanshidixit17@gmail.com; 2 . Department of Nursing Services, All India Institute of Medical Sciences, New Delhi, India. Email: anjaliratheee00@gmail.com. https://orcid.org/0009-0004-0460-4917 All India Institute Of Medical Sciences Department of Nursing Services All India Institute of Medical Sciences New Delhi India anjaliratheee00@gmail.com; 3 . College of Nursing, All India Institute of Medical Sciences, Raebareli, Uttar Pradesh, India. Email: surya.tiwari468@gmail.com. https://orcid.org/0000-0003-4718-0398 All India Institute Of Medical Sciences College of Nursing All India Institute of Medical Sciences Raebareli Uttar Pradesh India surya.tiwari468@gmail.com; 4 . College of Nursing, All India Institute of Medical Sciences, Rishikesh, Uttarakhand, India. Email: phalswaluma2828@gmail.com. Corresponding author. https://orcid.org/0000-0002-9801-1386 All India Institute Of Medical Sciences College of Nursing All India Institute of Medical Sciences Rishikesh Uttarakhand India phalswaluma2828@gmail.com; 5 . College of Nursing, All India Institute of Medical Sciences, New Delhi, India. Email: smitadas307@gmail.com. https://orcid.org/ 0000-0002-4157-601X All India Institute Of Medical Sciences College of Nursing All India Institute of Medical Sciences New Delhi India smitadas307@gmail.com

**Keywords:** emotional intelligence, sleep quality, clinical competence, nursing students, academic performance, nursing education, patient care., inteligencia emocional, calidad del sueño, competencia clínica, estudiantes de enfermería, rendimiento académico, formación en enfermería, atención al paciente., inteligência emocional, qualidade do sono, competência clínica, estudantes de enfermagem, desempenho acadêmico, educação em enfermagem, cuidados ao paciente**.**

## Abstract

**Objective.:**

This study aimed to investigate emotional intelligence (EI), sleep quality, and clinical competence among nursing students and identify factors associated with clinical competence.

**Methods.:**

This cross-sectional study included 199 pre-final and final-year nursing students from a college in Northern India. Data were collected using validated questionnaires measuring EI (Schutte Self-Report Emotional Intelligence Scale), sleep quality (Pittsburgh Sleep Quality Index), and clinical competence (Clinical Competence Questionnaire).

**Results.:**

Most students demonstrated high EI (56.3%) and good sleep quality (54.3%). EI was positively correlated with clinical competence (r=0.417, *p*<0.01) and negatively correlated with poor sleep quality (r=−0.173, p<0.05). Final-year students scored significantly higher on EI, clinical competence, and sleep disturbance than pre-final-year students. Multiple regression analysis identified academic year (β=0.208, *p*=0.002), EI (β=0.248, *p*<0.001), and sleep disturbance (β=−0.188, *p*=0.004) as significant predictors of clinical competence.

**Conclusion.:**

EI and sleep quality are important factors associated with the clinical competence of nursing students. Incorporating EI training and sleep hygiene education into nursing curricula may help improve students' clinical performance and prepare them for professional practice.

## Resumen

**Objetivo.:**

Investigar la inteligencia emocional (IE), la calidad del sueño y la competencia clínica entre los estudiantes de enfermería e identificar los factores asociados con la competencia clínica.

**Métodos.:**

Este estudio transversal incluyó a 199 estudiantes de enfermería de penúltimo y último año de una universidad del norte de la India. Los datos se recogieron mediante los cuestionarios validados: Escala de autoinforme de inteligencia emocional de Schutte, Índice de calidad del sueño de Pittsburgh y el Cuestionario de competencia clínica.

**Resultados.:**

Los estudiantes mostraron una IE alta (56.3%) y una buena calidad del sueño (54.3%). La IE se correlacionó positivamente con la competencia clínica (r=0.417, *p*<0.01) y negativamente con la pobre calidad del sueño (r=-0.173, *p*<0.05). Los estudiantes de último año puntuaron significativamente más alto en IE, competencia clínica y trastornos del sueño que los estudiantes de penúltimo año El análisis de regresión múltiple identificó el año académico (β=0.208, *p*=0.002), la IE (β=0.248, *p*<0.001) y las alteraciones del sueño (β=-0.188, *p*=0.004) como predictores significativos de la competencia clínica.

**Conclusión.:**

La IE y la calidad del sueño son factores importantes asociados a la competencia clínica de los estudiantes de enfermería. Incorporar la formación en IE y la educación en higiene del sueño en los planes de estudio de enfermería puede ayudar a mejorar el rendimiento clínico de los estudiantes y prepararlos para la práctica profesional.

## Resumo

**Objetivo.:**

Investigar a inteligência emocional (IE), a qualidade do sono e a competência clínica entre estudantes de enfermagem e identificar fatores associados à competência clínica.

**Métodos.:**

Este estudo transversal incluiu 199 estudantes de graduação e do último ano de enfermagem de uma universidade no norte da Índia. Os dados foram coletados usando questionários validados: Escala de Autoavaliação de Inteligência Emocional de Schutte, Índice de Qualidade do Sono de Pittsburgh e Questionário de Competência Clínica.

**Resultados.:**

Os estudantes apresentaram alta IE (56.3%) e boa qualidade do sono (54.3%). A IE foi positivamente correlacionada com a competência clínica (*r*=0.417, *p*<0.01) e negativamente correlacionada com a má qualidade do sono (*r*= -0.173, *p<0.05). Os estudantes do último ano pontuaram significativamente mais alto em IE, competência clínica e distúrbios do sono do que* os estudantes prefinais. A análise de regressão múltipla identificou o ano letivo (β =0.208, *p*=0.002), a IE (β =0.248, *p*<0.001) e os distúrbios do sono (β =-0.188, *p*=0.004) como preditores significativos da competência clínica.

**Conclusão.:**

A IE e a qualidade do sono são fatores importantes associados à competência clínica dos estudantes de enfermagem. A incorporação do treinamento em IE e da educação em higiene do sono aos currículos de enfermagem pode ajudar a melhorar o desempenho clínico dos estudantes e prepará-los para a prática profissional.

## Introduction

Emotional intelligence (EI), the ability to perceive, assess, communicate, regulate, and manage emotions, plays a crucial role in nursing. Nurses with high EI can communicate constructively, understand others' emotions, and improve their working relationships and performance in clinical settings.^1^ Although the importance of EI in nursing is well established, there are significant gaps in our understanding of how EI interacts with other factors that influence nursing students' clinical competence. Specifically, previous research has largely overlooked the complex interplay among EI, sleep quality, academic performance, and clinical competence in nursing students. This study aimed to address these gaps by examining these relationships.

Clinical competence refers to a nurse's ability to effectively apply knowledge, skills, and judgment to various healthcare situations.^2^ It encompasses factors such as interpersonal skills, patient management in multicultural settings and the ability to work well with others. Although academic performance has traditionally been used to assess nursing students' competence, it is not a strong predictor of clinical success.^3^ In contrast, clinical competence, which is closely linked to EI, appears to be more significant for clinical effectiveness.^4^ Studies have found relationships between EI and clinical performance, with emotionally intelligent students demonstrating better patient relationships, clinical reasoning, self-awareness, and adaptability.^5^ Additionally, this study examined two other factors that may affect nursing students' clinical competence: sleep quality and academic performance. Sleep quality, which includes factors such as sleep duration, efficiency, and disturbances, is another important factor that may affect nursing students' EI and performance.^6^ Poor sleep is common among college students and is associated with decreased academic performance, impaired concentration, and motivational issues.^7^

Academic performance, although not a strong predictor of clinical success, may play a role in overall clinical competence. It is typically measured through grades, test scores, and overall grade point averages. Understanding how academic performance interacts with EI, sleep quality, and clinical competence can provide valuable insights into developing targeted interventions to support nursing students’ success. The primary aim of this study was to investigate the levels of EI, sleep quality, and clinical competence among nursing students and identify the factors associated with clinical competence. By examining these factors together, this study sought to provide a more comprehensive understanding of the factors influencing nursing students' clinical competence. These findings may inform curriculum development and support strategies to enhance nursing education and practice, ultimately leading to better-prepared nursing graduates and improved patient care.

## Methods

Study design and setting. This descriptive cross-sectional study investigated EI, sleep quality, and clinical competence levels and identified factors associated with clinical competence among nursing students. Data were collected from August to October 2024 at a nursing college in Northern India.

Participants. Prefinal- and final-year bachelor’s nursing students were recruited using a convenience sampling technique. These years were chosen because students had gained sufficient clinical experience at this stage of their education. Students with a history of psychiatric illness, ongoing psychiatric treatment, sleep disorders, or the use of sleep medications were excluded.

Sample size. The sample size was calculated using the formula *n* = Z² P (1−P)/d², based on a 31.2% prevalence of clinical competence among nurses (2). The calculated sample size was 172 participants, with a 95% confidence interval and 7% precision. Accounting for a 10% response rate, the final sample size was adjusted to 190.

Ethical considerations. Before participation, the participants were informed of the aims and purpose of the study. Confidentiality and anonymity were ensured. Ethical approval was obtained from the Institutional Ethics Committee.

Data collection tools. This study utilized several data collection tools. A demographic information questionnaire was used to gather data on participants' age, religion, academic year, parental education, and family income. Academic performance was measured using the percentage score from the previous year.

**Instruments. (i) *Schutte Self-Report Emotional Intelligence Scale (SSREI):*** The SSREI is a five-point Likert scale ranging from 1 (strongly disagree) to 5 (strongly agree) and includes 33 items. EI was measured as the sum of the scores for all 33 items, with a score range of 33-165, where a score between 33 and 77 suggested a low EI level, a score between 78 and 121 indicated a moderate level, and a score between 122 and 165 denoted a high EI level.^8^ The reliability of the EI of nursing students was established using the test-retest method (r= 0.92). The SSREI has a reliability coefficient (Cronbach’s alpha=0.90) and validity (CVI=0.88) (8); **(ii) *Clinical Competence Questionnaire (CCQ)*:** The CCQ was used to evaluate the clinical competency of nursing students. It consists of 47 items that represent clinical competencies categorized as either nursing professional behavior (items 1-17), or related to clinical competency (items 17-47). The CCQ uses a 5-point Likert scale ranging from 1 (do not have a clue) to 5 (known in theory, competent in practice without supervision). The total score ranged from 47 to 235, with higher scores indicating good clinical competence. The Cronbach’s alpha for the 47-item Clinical Competence Questionnaire was 0.98;^9^**(iii)**

***Pittsburgh Sleep Quality Index (PSQI)*:** The PSQI was used to assess the sleep quality of students. It consists of 19 items based on sleep efficacy, sleep quality, sleep duration, perennial sleep efficiency, sleep disturbance, sleep medication use, and daytime dysfunction. The score for each component ranges from 0 to 3, with scores >5 representing poorer sleep quality. The total score ranges from 0 (easy sleep) to 21 (severe difficulty). The Cronbach’s alpha coefficient for the PSQI internal consistency reliability was 0.83.^10^ The Cronbach's alpha values for the SSREI, CCQ, and PSQI in this study were 0.914, 0.985, and 0.819, respectively.

Variables. The primary outcome was clinical competence, measured using the CCQ. The predictor variables included EI (SSREI), sleep quality (PSQI), academic performance, and demographic factors.

Statistical analysis. Prior to entry into the primary database, the information gathered was scrutinized for completeness and precision. Statistical evaluations were conducted using the SPSS software (version 26.0; IBM Corp., Armonk, N.Y., USA). Descriptive statistics, including percentages, means, and standard deviations, were computed to summarize the participants’ sociodemographic profiles, EI, sleep quality, and clinical competence scores. Cronbach’s alpha was used to assess the reliability of the scale. Participants were categorized based on their clinical competence scores through a two-step cluster analysis employing the log-likelihood distance measure and Schwarz's Bayesian Information Criterion (BIC) to ascertain the optimal cluster count. Inferential statistics were calculated according to the central limit theorem (CLT). Differences in the mean EI, sleep quality, and clinical competence levels according to academic year and performance were analyzed using an independent t-test and one-way analysis of variance (ANOVA), respectively. Levene's test was used to evaluate the homogeneity of variance using one-way ANOVA. Cohen’s d effect sizes were calculated for significant t-test results to quantify the magnitude of differences between the groups. Cohen's d values of 0.2, 0.5, and 0.8 were considered small, medium, and large effect sizes, respectively. For the ANOVA results, eta-squared (η2) was calculated to estimate the proportion of variance in the dependent variable explained by the independent variable. η2 values of 0.01, 0.06, and 0.14 were interpreted as small, medium, and large effect sizes, respectively. Spearman's rho was used to assess correlations between dependent variables. Univariate regression analysis was used to identify potential associations among sociodemographic factors, study variables, and clinical competence. Multivariate linear regression analyses were conducted to identify predictors of clinical competence, incorporating variables with *p*-values <0.2 into the model. All statistical tests were two-tailed, with statistical significance set at *p*<0.05.

## Results

### Socio-demographic profile of participants

The study included 199 nursing students (response rate:81.84%), with the majority being aged 22-23 years (64.3%) and unmarried (97.5%). Over half of the participants (55.3%) were in the pre-final year of their nursing programs. Academic performance varied, with 51.8% scoring between 70% and 79% in their last professional examination. Most participants (81.9%) reported an interest in pursuing a career in nursing (Table 1).

**Table 1:** Socio-demographic profile of 199 nursing students

Variable	Categories	Frequency (%)
Age (years)	19-21 22-23 23-25	48 (24.1) 128 (64.3) 23 (11.6)
Religion	Hindu Others	180 (90.5) 19 (9.5)
Marital status	Unmarried Married	194 (97.5) 5 (2.5)
Academic year	Pre-final Final	110 (55.3) 89 (44.7)
Family monthly income (INR)	Below 25,000 25,000-50,000 50,000-75,000 Above 75,000	39 (19.6) 73 (36.7) 50 (25.1) 37 (18.6)
Marks in the last professional examination	Below 60% 60-69% 70-79% > 80%	12 (6.0) 78 (39.2) 103 (51.8) 6 (3.0)
Interested in nursing profession	No Yes	36 (18.1) 163 (81.9)

Classification of EI, Sleep Quality, and Clinical Competence. The mean EI, global PSQI, and CCQ scores were 121.92±14.57, 5.85 ± 2.94, and 178.26 ± 39.58, respectively. Most of the study participants had high EI (56.3%) and good sleep quality (54.3%). Cluster analysis was chosen to classify clinical competence because of its suitability for identifying naturally occurring patterns and offering insights into groupings based on shared characteristics. The silhouette measures of cohesion and separation were fair. Cluster 1 (55.3%, n=110) had the highest mean scores and was labeled as the good clinical competence group, while Cluster 2 (44.7%, n=89) had lowest mean scores and were labeled as poor clinical competence (Table 2).

**Table 2:** Classification of Emotional Intelligence, Sleep Quality, and Clinical Competence Scores

Variables (Score)	Frequency (%)	Mean ± SD
SSEIT High (122-165) Moderate (78-121) Low (33-77)	112 (56.3) 85 (42.7) 2 (1.0)	121.92±14.57
CCQ Good Poor	110 (55.3) 89 (44.7)	178.26±39.58
Global PSQI Good (0-5) Poor (6-21)	108 (54.3) 91 (45.7)	5.85±2.94

Abbreviations: SSEIT- Schutte Self Report Emotional Intelligence Test; PSQI- Pittsburgh Sleep Quality Index; CCQ- Clinical Competence Questionnaire

Correlations between study variables. The major findings revealed significant positive correlations between EI and overall clinical competence (*r*=0.417, *p*<0.01), as well as between EI and the individual domains of clinical competence. EI also showed a negative correlation with the global PSQI score (*r*=−0.173, *p*<0.05), indicating better sleep quality with higher EI. Poor sleep quality was negatively correlated with overall clinical competence (r=−0.148, *p*<0.05) and its domains. Specifically, the use of sleep medication (*r*=−0.141, *p*<0.05) and daytime dysfunction (r=−0.190, *p*<0.01) were significantly negatively correlated with overall clinical competence. Strong positive correlations were observed between the clinical competence domains, particularly between core and advanced nursing skills (r=0.932, *p*<0.01).

Mean differences across academic year. Table 3 shows the mean score differences in the study variables between pre-final and final-year students. Final-year students scored significantly higher than pre-final-year students in EI (t=−3.443, *p*=0.001, Cohen’s d= -0.4909), global PSQI (t=−2.599, *p*=0.010, Cohen’s d= -0.3705), and all clinical competence domains. Notably, advanced nursing skills (t=−4.153, *p*<0.001, Cohen’s d= -0.5921) and core nursing skills (t=−3.989, *p*<0.001, Cohen’s d= -0.5688) had medium effect sizes.

**Table 3:** Emotional Intelligence, Sleep Quality, and Clinical Competence Scores by Academic Year

	Pre-final year	Final year	t-value	***p*-value**	Cohen’s d
SSEIT	118.81±15.91	125.78±11.70	-3.443	0.001	-0.4909
Subjective sleep quality	0.80±0.73	1.13±0.77	-3.115	0.002	-0.4441
Sleep latency	1.15±0.83	1.35±0.90	-1.565	0.119	-0.2231
Sleep duration	1.18±0.52	1.21±0.48	-0.435	0.664	-0.0620
Sleep efficiency	0.45±0.65	0.51±0.65	-0.544	0.587	-0.0775
Sleep disturbance	1.01±0.61	1.09±0.57	-0.949	0.344	0.1353
Use of sleep medication	0.22±0.56	0.29±0.74	-0.798	0.426	-0.1138
Daytime dysfunction	0.55±0.74	0.87±0.90	-2.646	0.009	-0.3773
Global PSQI score	5.37±2.72	6.45±3.11	-2.599	0.010	-0.3705
Nursing professional behaviour	54.88±14.33	60.66±11.80	-3.057	0.003	-0.4358
Skill competence: general	46.88±13.08	52.48±9.86	-3.342	0.001	-0.4765
Skill competence: core nursing skills	45.68±12.77	52.13±9.28	-3.989	<0.001	-0.5688
Skill competence: advance nursing skills	21.46±5.59	24.53±4.60	-4.153	<0.001	-0.5921
Overall CCQ	168.91±42.47	189.81±32.36	-3.829	<0.001	-0.5459

Abbreviations: SSEIT- Schutte Self Report Emotional Intelligence Test; PSQI- Pittsburgh Sleep Quality Index; CCQ- Clinical Competence Questionnaire

Mean differences across Academic Performance. Significant differences were observed in EI (F=2.774, *p*=0.043, η2=0.041) and sleep duration (F=4.902, *p*=0.003, η2=0.070) across academic performances. Higher-performing students (>80%) also showed better scores for professional nursing behavior (F=3.786, *p*=0.011, η2=0.055) and overall clinical competence (F=3.026, *p*=0.031, η2=0.044) (Table 4).

**Table 4:** Emotional Intelligence, Sleep Quality, and Clinical Competence Scores by Academic Performance

	Below 60%	60-69%	70-79%	> 80%	F-value	***p*-value**	eta-square
SSEIT	115.17±12.29	119.45±15.35	124.50±13.58	123.50±18.38	2.774	0.043	0.041
Sleep latency	1.08±0.99	0.92±0.76	0.97±0.76	0.67±0.51	0.444	0.722	0.007
Sleep duration	1.17±0.83	1.44±0.97	1.17±0.84	0.17±0.40	4.902	0.003	0.070
Sleep efficiency	1.25±0.45	1.09±0.51	1.28±0.49	1.00±0.63	2.497	0.061	0.037
Sleep disturbance	0.83±0.57	0.47±0.65	0.44±0.66	0.50±0.54	1.311	0.272	0.020
Use of sleep medication	1.17±0.71	1.09±0.58	1.02±0.59	0.67±0.51	1.181	0.318	0.018
Daytime dysfunction	0.00±0.00	0.27±0.63	0.25±0.68	0.50±0.83	0.912	0.436	0.014
Sleep latency	0.442±0.79	0.68±0.87	0.71±0.77	1.17±1.32	1.100	0.350	0.017
Global PSQI score	5.92±2.35	5.96±2.35	5.83±2.92	4.67±1.36	0.359	0.783	0.005
Nursing professional behaviour	46.42±18.40	56.55±13.08	59.55±12.60	55.67±15.44	3.786	0.011	0.055
Skill competence: general	40.08±15.64	49.01±11.39	51.05±11.70	44.33±11.02	3.544	0.016	0.052
Skill competence: core nursing skills	42.42±16.30	48.31±11.66	49.50±11.27	48.33±10.00	1.327	0.267	0.020
Skill competence: advance nursing skills	19.50±7.25	22.76±5.20	23.24±5.33	23.50±1.87	1.789	0.151	0.027
Overall CCQ	148.42±55.98	176.63±37.89	183.34±37.77	171.83±34.42	3.026	0.031	0.044

Abbreviations: SSEIT- Schutte Self Report Emotional Intelligence Test; PSQI- Pittsburgh Sleep Quality Index; CCQ- Clinical Competence Questionnaire

Factors Associated with Clinical Competence. The multiple linear regression model identified several significant predictors of clinical competence (R^2^=0.239; adjusted R^2^=0.207). Final-year students had higher clinical competence scores than pre-final-year students (β=0.208, *p*=0.002). EI was a strong positive predictor of clinical competence (β=0.248, *p*<0.001). Higher levels of sleep disturbance negatively affected clinical competence (β=−0.188, *p*=0.004). Other variables, such as family income, marks on the last professional examination, and interest in the nursing profession, were not significant predictors in the adjusted model (Table 5).

**Table 5:** Multiple Linear Regression Analysis of Factors Associated with Clinical Competence

Variables	CCQ			
	Unadjusted estimates			Adjusted estimates (R^2^ = 0.239)		
	*B*	95% CI	*p-value*	*B*	95% CI	*p-value*	*β*
Age	0.389	-9.112, 9.890	0.936	-	-	-	-
Religion	4.197	-14.673, 23.067	0.661	-	-	-	-
Marital status	-11.752	-47.163, 23.660	0.514	-	-	-	-
Academic year	20.900	10.135, 31.665	<0.001**	16.511	6.092, 26.930	0.002*	0.208
Monthly family income	5.970	0.510, 11.431	0.032*	4.943	-0.020, 9.907	0.051	0.126
Marks in the last professional examination	9.390	1.038, 17.741	0.028*	4.387	-3.475, 12.249	0.272	0.073
Interested in nursing profession	16.828	2.611, 31.046	0.021*	2.647	-10.937, 16.231	0.701	0.026
SSEIT	0.953	0.595, 1.310	<0.001**	0.673	0.301, 1.046	<0.001**	0.248
Sleep latency	0.864	-6.355, 8.084	0.236	-	-	-	-
Sleep duration	0.643	-5.738, 7.024	0.843	-	-	-	-
Sleep efficiency	-3.232	-14.144, 7.680	0.560	-	-	-	-
Sleep disturbance	-13.104	-21.358, -4.850	0.002*	-11.317	-18.979, -3.654	0.004*	-0.188
Use of sleep medication	-9.637	-18.853, -0.421	0.041*	-3.679	-12.937, 5.579	0.434	-0.055
Daytime dysfunction	-9.370	-17.837, -0.904	0.030*	-5.314	-13.846, 3.218	0.221	-0.087
Sleep latency	-2.671	-9.315, 3.974	0.429	-	-	-	-
Global PSQI score	-1.698	-3.569, 0.174	0.075	-	-	-	-

B= Unstandardized coefficient; β=Standardized coefficient, “Global PSQI was removed from adjusted model due to multicollinearity

Abbreviations: SSEIT- Schutte Self Report Emotional Intelligence Test; PSQI- Pittsburgh Sleep Quality Index; CCQ- Clinical Competence Questionnaire

## Discussion

This study examined the relationships between EI, sleep quality, academic performance, and clinical competence in nursing students. These findings provide valuable insights into the complex interactions and their implications for nursing education and practice. Most students (56.3%) demonstrated high EI levels, with final-year students scoring significantly higher than pre-final-year students. This suggests that EI may improve over the course of nursing education,^11^ possibly because teaching methods enhance EI skills during clinical training. These findings align with previous research showing a moderate positive correlation between EI and effective clinical teaching.^12^ High EI scores indicate that many students in this study were adept at managing daily challenges and regulating their own and others’ emotions. However, it is worth noting that other studies found average EI scores among nursing students,^13^ highlighting the need for further research in this area.

Sleep is a vital aspect of life, particularly for students who are highly susceptible to sleep-related issues. In contrast to some previous studies on medical students that found poor sleep quality rates of 19-33.8% (14^,15)^, this study found that 45.7% of nursing students experienced poor sleep quality. This discrepancy may be due to various factors influencing sleep quality, including socioeconomic status, academic demands, and health.^16^ Interestingly, final-year students had higher PSQI scores than pre-final-year students, indicating poorer sleep quality among more advanced students. The improvement in EI scores from the pre-final to the final year indicates that nursing education may naturally enhance EI skills over time. However, the concurrent decline in sleep quality among final-year students raises concerns about the potential trade-offs between academic demands and personal wellbeing. Senior nursing students in this study displayed high levels of clinical competence, which differs from previous research that found moderate performance levels.^17^ Our findings are encouraging and may reflect the effectiveness of nursing programs in developing students' clinical skills over time.

We found a significant positive correlation between EI and overall clinical competence. This finding supports the idea that students with higher EI are better equipped to handle the emotional demands of clinical practice, potentially leading to improved patient care and outcomes. These results are consistent with those of other studies, suggesting a positive relationship between EI and nursing performance.^18^ However, it is important to note that some studies have shown weak or no significant relationship between EI and clinical performance.^19^ The significant relationship between EI and sleep quality in our study is consistent with previous studies.^20^ Although sleep duration did not significantly correlate with EI, overall sleep quality was associated with higher EI scores. This aligns with studies showing that factors such as mood and emotions affect sleep patterns and that sleep deprivation can impair emotional recognition.^21^ Our study found a strong relationship between EI and academic performance, supporting previous research that has shown similar correlations among nursing and other healthcare students.^22^ This suggests that developing EI skills may have benefits beyond clinical practice and potentially improve overall academic success of students.

Although this study did not find a significant association between PSQI scores and academic performance, a negative correlation was observed between daytime dysfunction and clinical competence. The negative correlation between sleep disturbance and clinical competence highlights the often-overlooked effect of sleep quality on nursing performance. Poor sleep quality, particularly among senior students, may hinder their ability to perform effectively in clinical settings. This aligns with other studies showing that students with better clinical performance tend to have better sleep quality.^23^ The lack of an association between overall sleep quality and academic performance contrasts with previous research,^24^ suggesting the need for further studies. The multiple linear regression model identified several significant factors associated with clinical competence, and final-year students had higher clinical competence scores than pre-final-year students. The EI is a strong positive predictor of clinical competence. Higher levels of sleep disturbance negatively affected their clinical competence. In contrast, Park and Chung found that older nursing students performed better in clinical practice.^25^ However, our findings were not conclusive. Interestingly, family income and interest in nursing were not significant predictors in the adjusted model. The study also revealed that academic performance, although important, may not be the strongest predictor of clinical competence in the field. This challenges traditional assumptions regarding the relationship between academic success and clinical skills, suggesting that a more holistic approach to nursing education is necessary.

This study has several notable strengths, including its comprehensive approach to examining multiple interrelated factors, use of validated measurement tools, adequate sample size that met the calculated requirements, and diverse statistical analyses. The consideration of multiple predictors in the regression analysis allowed for a nuanced understanding of the factors influencing clinical competence.

Limitations and Future directions. The study conducted at a single nursing college in Northern India has several limitations that should be considered. The cross-sectional design and reliance on self-reported data from a specific group of nursing students limit the generalizability of the findings and prevent the establishment of causal relationships. The subjective assessment of sleep quality and the potential influence of unmeasured confounding variables further constrained the scope of this study. To address these limitations and advance the field, future research should focus on multicenter longitudinal studies that incorporate objective sleep measures and a more diverse student population. Expanding research to include mixed-method approaches, intervention studies, and cultural considerations would provide a more comprehensive understanding of the factors influencing clinical competence. Additionally, integrating technology, exploring mentors’ perspectives, and conducting comparative studies across healthcare professions may yield valuable insights.

Practical implications. This study has several practical implications for nursing education and practice. Incorporating EI training into nursing curricula, focusing on skills such as self-awareness, empathy, and emotion regulation, is crucial. Workshops and mentoring programs can enhance students' EI throughout their academic journeys. Educating students about the importance of good sleep hygiene and its impact on clinical performance is essential, along with implementing stress management and relaxation techniques to help improve sleep quality. Adjusting clinical schedules to allow better sleep patterns, especially among senior students, should be considered. Developing targeted interventions to improve clinical competence, particularly for students with lower EI scores or poor sleep quality, and incorporating more hands-on clinical experiences and simulations can build competence over time. It is recommended to implement regular assessments of EI, sleep quality, and clinical competence to identify at-risk students early and provide individualized support and resources based on these assessments. Integrating EI concepts and skills throughout nursing programs and emphasizing the connections between EI, sleep quality, and clinical performance in coursework are important. Training nursing educators on the importance of EI and sleep quality in student performance and equipping faculty with the tools to assess and support students in these areas are crucial.

Conclusions. This study provides valuable insights into the complex relationships between EI, sleep quality, academic performance, and clinical competence in nursing students. These findings highlight the importance of EI and sleep quality in predicting clinical competence, suggesting potential areas for intervention in nursing education. By addressing these factors, nursing programs may enhance students' clinical performance and better prepare them for the challenges of professional practice. Future research should build on these findings to develop and evaluate targeted interventions that can improve nursing students' EI, sleep quality, and ultimately, their clinical competence.
